# Residual Stresses in Ribbed Reinforcing Bars

**DOI:** 10.3390/ma17010026

**Published:** 2023-12-20

**Authors:** Tobias Robl, Patrick Hegele, Christian Krempaszky, Ewald Werner

**Affiliations:** Institute of Materials Science, Department of Materials Engineering, TUM School of Engineering and Design, Technical University of Munich, 85748 Garching, Germany; patrick.hegele@tum.de (P.H.); krempaszky@tum.de (C.K.)

**Keywords:** reinforcing steel, manufacturing process, stress concentration, residual stresses, constitutive modeling

## Abstract

Ribbed reinforcing bars (rebars) are used for the reinforcement of concrete structures. In service, they are often subjected to cyclic loading. In general, the fatigue performance of rebars may be influenced by residual stresses originating from the manufacturing process. Knowledge about residual stresses in rebars and their origin, however, is sparse. So far, residual stress measurements are limited to individual stress components, viz., to the non-ribbed part of the rebar surface. At critical points of the rebar surface, where most of the fatigue cracks originate, i.e., the foot radius regions of transverse ribs, the residual stress state has not yet been investigated experimentally. To extend the knowledge about residual stresses in rebars within the scope of this work, residual stress measurements were carried out on a rebar specimen with a diameter of 28 mm made out of the rebar steel grade B500B. In addition, numerical simulations of the TempCore^TM^ process were carried out. The results of the experimental investigations show tensile residual stresses in the core and the transition zone of the examined rebar specimen. Low compressive residual stresses are measured at the non-ribbed part of the rebar surface, while high compressive residual stresses are present at the tip of the transverse ribs. The results of the numerical investigations are in reasonable accordance with the experimental results. Furthermore, the numerical results indicate moderate tensile stresses occurring on the rebar surface in the rib foot radius regions of the transverse ribs. High stress gradients directly beneath the rebar surface, which are reported in the literature and which are most likely related to a thin decarburized surface layer, could be reproduced qualitatively with the numerical model developed.

## 1. Introduction

**Figure 1:**
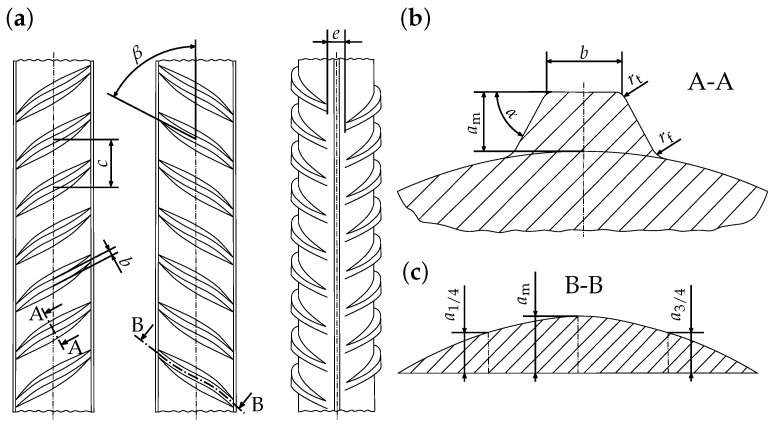
Idealized geometry of rebar steel grade B500B with longitudinal ribs according to DIN-488-2 [1]: (**a**) overall view, (**b**) cross-section of transverse rib and (**c**) longitudinal section of transverse rib.

Reinforcing bars are cylindrical steel bars (Figure 1) used for the reinforcement of concrete structures [1,2]. The most common rebar steel grade in the German market is denoted as B500B. A typical manufacturing route is the so-called TempCore^TM^ process, during which the rebar is hot-rolled in fully austenitic state and then partially quenched. After partial quenching, the rebar is cooled down in air to room temperature. During hot-rolling, ribs are formed on the rebar surface. Due to partial quenching, the rim of the rebar (and the transition zone) transforms into lath martensite (and bainite) up to a certain hardening depth, while the core stays austenitic. During cooling in air, the austenite in the core transforms into ferrite and pearlite. At the same time, the martensitic rim is reheated and tempered by the heat from the core. By adjusting the heat-treatment parameters, e.g., by varying the quenching time, the TempCore^TM^ process allows the production of rebars of different strengths and ductilities [3,4,5].

From this heat-treatment, residual stresses originate. They are caused by the asynchronous shrinkages of the outer rim, the transition zone and the core region as well as by the transformation strains accompanying phase transformations both during quenching and cooling in air [6]. In service, these residual stresses are superimposed by mechanical stresses caused by (often cyclic) mechanical loads. While compressive residual stresses on the rebar surface potentially favor an improved fatigue performance, tensile residual stresses may lead to an earlier failure of the reinforcement [6]. Despite this fact, and the obvious potential it offers in terms of an increased fatigue performance, knowledge about residual stresses in rebars and their origin is sparse.

Experimental approaches to determine residual stresses in rebars are limited to individual stress components, viz., to the non-ribbed part of the rebar surface after complete cooling [7,8,9,10,11]. Furthermore, the results of these experimental investigations are partly contradictory. Also, in particular critical regions of the rebar surface, where most of the fatigue cracks form, i.e., the foot radius regions of the transverse ribs, residual stresses have not yet been investigated experimentally. A numerical model suggested by the authors of this study [3] overestimates the residual stress level in rebars quantitatively because presumably important effects have not been considered, such as transformation-induced plasticity (TRIP) and tempering of the martensitic rim during cooling in air.

In order to extend the knowledge about residual stresses in rebars, in this work residual stress measurements were carried out on a rebar specimen. The results of these measurements were compared to the results of numerical simulations of the TempCore^TM^ process, whereas the numerical model used was an extended version of the model of Robl et al. [3]. In the extended model, tempering of martensite and TRIP were considered. In this manuscript, the theoretical background regarding both effects is provided in Section 2. Also in Section 2, an overview is given about the residual stress measurements, which have already been carried out by other researchers. The procedures and results of the current residual stress measurements are presented in Section 3. Furthermore, the procedures and the results of further experimental standard investigations are presented, which were carried out to justify certain modeling assumptions and to verify the results of the numerical simulations. Since the main focus of this manuscript is on the numerical simulations, a detailed explanation of the extended modeling approach is given in Section 4.1. The simulation results are presented and discussed in Section 4.2. The numerical results are compared to the results from Section 3 and to experimental results from the literature.

## 2. Theory

### 2.1. Tempering of Martensite

Tempering describes the heat-treatment of martensite in steels with the purpose to modify mechanical properties. During tempering, the yield strength of martensite decreases, whereas its ductility increases. Tempering of martensite is also accompanied by a change in the specific volume. During tempering, the martensitic microstructure approaches equilibrium, which, however, is never reached in technical heat-treatments [12].

In general, the tempering of martensite in plain carbon steels can be distinguished into four distinct stages [12]. These stages are:

- Stage 1: precipitation of ε-iron carbide (Fe_2.4_C) and partial loss of tetragonality via diffusion of carbon atoms to dislocations and grain boundaries;
- Stage 2: decomposition of retained austenite;
- Stage 3: dissolution of the ε-iron carbides formed in stage 1, formation of cementite (Fe_3_C) and full loss of tetragonality in martensite;
- Stage 4: coarsening and spheroidization of cementite and recrystallization of ferrite.

In plain carbon steels with a carbon content of 0.2 wt.% or lower, i.e., the rebar steel grade B500B, no retained austenite is formed during quenching. Furthermore, no ε-iron carbide is formed during the first stage of tempering. Both can be attributed to the low carbon content of the steel grade [12]. As plain carbon steels with low carbon content (0.2 wt.% or lower) exhibit high martensite start temperatures [3,11], the majority (up to 90%) of the carbon atoms segregates to dislocations and lath boundaries during quenching at comparatively low cooling rates [12,13]. At very low cooling rates, also the precipitation of cementite may occur. Therefore, martensite with a carbon content of 0.2 wt.% or lower shows no significant tetragonality after quenching in the course of most technical quenching processes.

Due to the very high cooling rates during the partial quenching step of the TempCore^TM^ process, neither the diffusion of carbon atoms to dislocations and grain boundaries nor the precipitation of cementite take place. Both most likely take place during reheating of the martensitic rim after partial quenching, which also accounts for the spheroidization of cementite. However, due to reheating no recrystallization of the microstructure takes place. This is due to rather short tempering times at high temperatures.

### 2.2. Transformation-Induced Plasticity

Transformation-induced plasticity (TRIP) can be explained as the significantly increased plastic strain, which occurs during a phase transformation under an externally applied load. Plastic deformation even may occur due to “an externally applied load for which the corresponding equivalent stress is small compared to the […] yield stress of the material” [14,15].

For diffusive phase transformations in steel, TRIP is associated to the Greenwood– Johnson effect [16]. During phase transformation, the transformation volume strain accompanying the decomposition of austenite into bainite, pearlite and ferrite leads to additional plastic straining of the austenite matrix. If the phase transformation takes place under externally applied loads, this plastic straining has a preferred spatial orientation and therefore contributes to the macroscopic phenomenon of TRIP [17]. For martensitic phase transformations in steel, the Greenwood–Johnson effect occurs not only due to the transformation volume strain accompanying the decomposition of austenite into martensite but also to the shear strain associated with the transformation [15]. Additionally, TRIP is related not only to the Greenwood–Johnson effect for martensitic phase transformations in steel but also to the Magee effect [16,18]. The Magee effect occurs due to the formation of martensite variants with a preferred crystallographic orientation. A preferred crystallographic orientation of martensite variants develops if a macroscopic stress is applied during martensite formation. Due to the formation of martensite variants with a preferred crystallographic orientation, the shear strain associated to the formation of individual martensite variants on the microscale does not average out on the macroscale [15,19,20].

### 2.3. Residual Stress Measurements on Rebars

Within the scope of residual stress measurements on rebars in the literature, X-ray diffraction techniques and cut-compliance methods have been applied. Residual stress measurements on rebars from the literature are limited to individual stress components, viz., to the non-ribbed part of the rebar surface. The results of these measurements are also contradictory to some extent. At particular critical regions of the rebar surface where most of the fatigue cracks form, i.e., in the rib foot radius regions of transverse ribs, the residual stress state has not been investigated yet.

**Figure 2:**
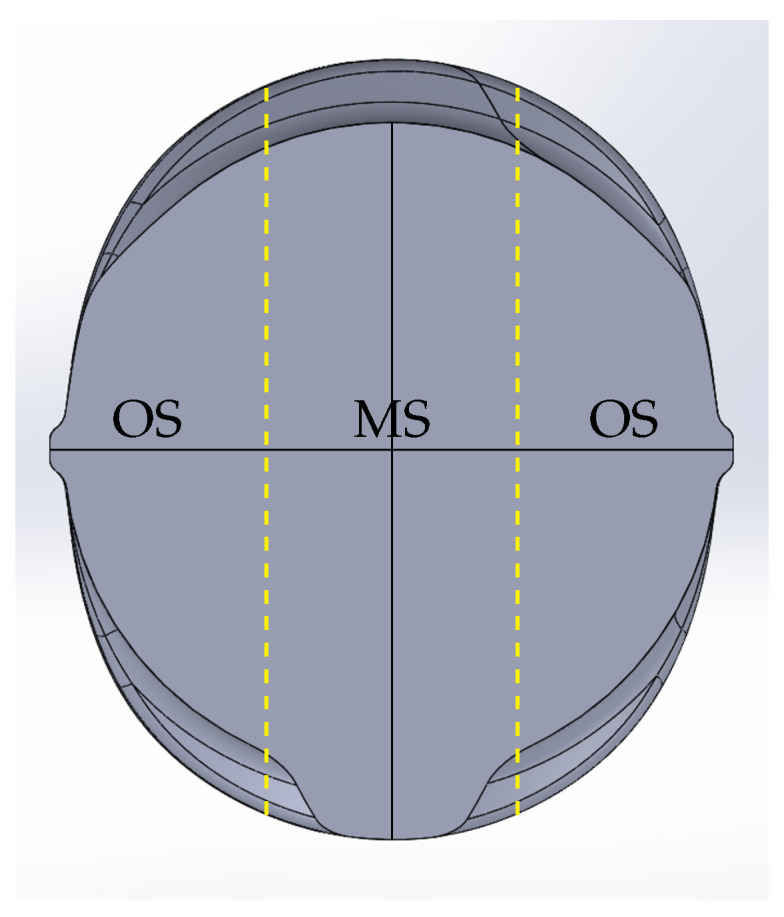
Idealized CAD-representation of a rebar (cross-section): middle segment used by Volkwein et al. [7] for residual stress measurements indicated by MS, outer segments indicated by OS.

- On the surface of a rebar with a diameter of d=28 mm, Hameed et al. [11] determined compressive axial and compressive tangential residual stresses of about −10 MPa and −40 MPa, respectively, between alternating ribs. At a depth of 0.3 mm below the rebar surface, Hameed et al. [11] determined axial and tangential compressive residual stresses of about −90 MPa and about −105 MPa, respectively. For the residual stress measurements, Hameed et al. [11] used an X-ray diffraction technique.
- On the surface of rebars with diameters of d={16,24,32} mm, Zheng et al. [8] determined axial compressive residual stresses between −80 MPa and −90 MPa by using a cut-compliance method.
- Volkwein et al. [7] determined axial compressive residual stresses of approximately −40 MPa on the surface of a first rebar with a diameter of d=28 mm. On the surface of a second rebar with the same diameter, which was produced by a different manufacturer, they determined axial tensile residual stresses of approximately 10 MPa. In the transition zone, Volkwein et al. [7] determined tensile residual stresses of approximately 45 MPa and 40 MPa, respectively. In the core, the authors determined compressive residual stresses of approximately −30 MPa and −45 MPa, respectively. For the residual stress measurements, Volkwein et al. [7] used a cut-compliance method. At first, the authors partitioned the rebar specimens each into two outer segments and one middle segment (see Figure 2). Then, they carried out the residual stress measurements only on the middle segment.
- Rocha et al. [9] measured axial compressive residual stresses in the non-ribbed region of the surface of a rebar with a diameter of d=16 mm. Between alternating ribs, they determined axial compressive residual stresses within the range of −48 MPa to −147 MPa and between parallel ribs within the range of −26 MPa to −61 MPa. At a depth of 0.05 mm, Rocha et al. [9] measured axial tensile residual stresses of max. 50 MPa. Within the depth range from 0.05 mm to 2 mm, the authors measured axial tensile residual stresses as well. The maximum value of the axial tensile residual stresses in this depth range was 120 MPa. For the measurements on the rebar surface, Rocha et al. [9] used an X-ray diffraction technique and for the measurements beneath the surface, they used a cut-compliance method.

The results of Hameed et al. [11] and Rocha et al. [9] showed high stress gradients in a thin surface layer (approximately 30 µm to 50 µm) directly beneath the rebar surface. However, these stress gradients were not captured by the residual stress measurements by Zheng et al. [8] and Volkwein et al. [7], most probably because both used cut-compliance methods with a too coarse resolution.

## 3. Experimental Investigations

To extend the knowledge about residual stresses in rebars, residual stress measurements were carried out on one rebar specimen with a diameter of d=28 mm fabricated from the rebar steel grade B500B. To justify specific modeling assumptions and to verify the results of the numerical simulations (Section 4), further experimental investigations were carried out on the same rebar specimen. These investigations included standard testing procedures like hardness measurements and the analysis of the microstructure by use of micrographs and EBSD measurements. The specimen used was manufactured by the same manufacturer as the first specimen examined by Volkwein et al. [7]. It was from the same batch as the specimens investigated by Hameed et al. [11] and Rappl et al. [21].

### 3.1. Residual Stress Measurements

Residual stress measurements on the rebar specimen were carried out on the rebar surface as well as in the core and in the transition zone. To carry out the residual stress measurements in the core and the transition zone, a representative, periodic section, i.e., a periodic unit cell, was extracted from the rebar specimen. For the residual stress measurements, the device Xstress 3000 with a G2R goniometer from the manufacturer stresstech (Vaajakoski, Finland) was used. The device was equipped with a Cr tube (operated at 30 kV and 8 mA), a 2 mm pinhole collimator and two position-sensitive detectors with V-filters in the secondary beampath. The instrument operated in modified χ-geometry, where χ is the angle between the normal to the specimen surface and the plane containing the incident beam as well as the vector normal to the diffracting lattice plane. The instrument was set up to record the {211}-bcc peak for various χ-angles between −45° and 45°. For the evaluation of the measurement results, the sin^2^Ψ method was applied relying on the diffraction elastic constants $E_{211}=211$ GPa and $\nu_{211}=0.3$ [22]. The $K_{\alpha 1}$ peak positions used for this method were determined by fitting pseudo-Voigt functions separately to the $K_{\alpha 1}$ and $K_{\alpha 2}$ components of the recordings [23].

#### 3.1.1. Residual Stress Measurements on the Surface

**Figure 3:**
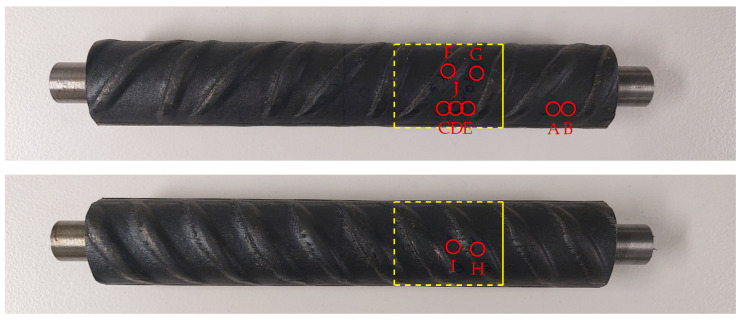
Examined rebar specimen with locations A-J for the residual stress measurements on the rebar surface ((**top**) rebar side with alternating ribs, (**bottom**) rebar side with parallel ribs): the periodic unit cell extracted from the rebar specimen, which was used for the residual stress measurements in the core and in the transition zone of the rebar, is highlighted in yellow (projection of the measurement surface for the residual stress measurements in the core and in the transition zone depicted as solid line).

Residual stresses in the axial and tangential directions were determined on the rebar surface. The exact measurement locations are shown in Figure 3. The results of the measurements are listed in Table 1.

From the results the following observations can be made: the measured residual stress level on the rebar surface was far below the yield stress of the martensitic rim for all measurement locations. Between alternating and parallel ribs, only slight compressive residual stresses can be reported in the axial direction. In the tangential direction, compressive residual stresses with a maximum value of −44 MPa were determined. The mean value of all measurement values, which were determined between the parallel and alternating ribs, was −6 MPa in the axial direction and −33 MPa in the tangential direction. At the tip of transverse ribs, high compressive residual stresses were determined. The maximum value at the tip of the ribs was −77 MPa in the axial and −158 MPa in the tangential direction. Both values were determined on the rebar side with alternating ribs.

#### 3.1.2. Residual Stress Measurements in the Core and the Transition Zone

**Figure 4:**
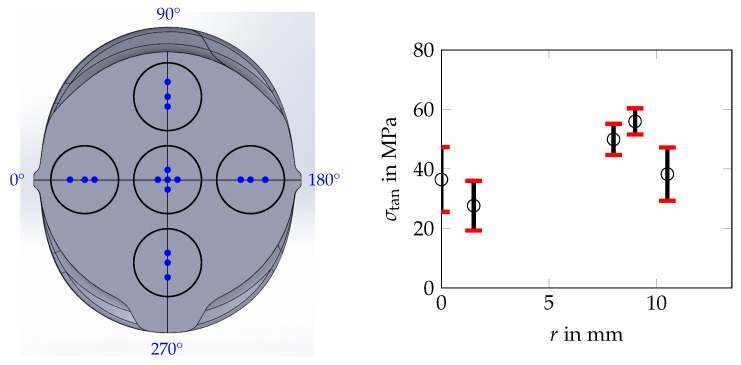
Residual stress measurements in the core and the transition zone of the extracted unit cell: electropolishing spots and selected evaluation locations highlighted in black and blue on an idealized CAD representation of the rebar specimen used (**left**) as well as the results of the residual stress measurements, i.e., tangential stress component, $\sigma_{\tan}$, as a function of the radius, *r* (**right**).

Also in the core and transition zone of the rebar, residual stress measurements were carried out. For these measurements, a periodic unit cell was extracted from the rebar specimen (see Figure 3). The extraction of the unit cell was performed by wire electric discharge machining (WEDM). The residual stress measurements were carried out on one of the front surfaces of this unit cell and were restricted to the tangential stress component, $\sigma_{\tan}$. The exact measurement locations are shown in Figure 4 (left).

The surface layer, which was influenced by WEDM during the extraction of the unit cell from the rebar specimen, was removed by grinding and electropolishing. Grinding was carried out in three steps with grade 320-, grade 1000- and grade 4000-grit sandpapers. Electropolishing was carried out with the electrolytic polisher and etcher Kristall 650 from the manufacturer ATM (Mammelzen, Germany), and etchant Elektrolyt K1 from ATM was used. With the electrolytic polisher and etcher, five individual, non-overlapping electropolished spots were prepared (spot diameter approximately 7 mm, etching current 2 A, etching time 30 s). One electropolished spot was located in the core of the rebar and the four other spots were located in the transition zone (see Figure 4 (left)). In the electropolished spot at the center, eight residual stress measurements were carried out (four at a radius position of 0 mm and four at a radius position of 1.5 mm). At each of the electropolished spots within the transition zone, three residual stress measurements were carried out (four at a radius of 8 mm, four at a radius of 9 mm and four at a radius of 10.5 mm). The results of the measurements are given in Table 2. Assuming an axisymmetric stress state in the core and transition zone of the specimen (see also Section 4.2.2), the mean values (MVs) of the measured residual stress values and the associated standard deviations (STDs) were determined for each measurement radius, i.e., for 0.0 mm, 1.5 mm, 8.0 mm, 9.0 mm and 10.5 mm. Six values were excluded from this analysis as the micrographs of the associated measurement locations showed scratches. The mean values and standard deviations are listed in Table 2 and are shown in Figure 4 (right).

The results of the evaluation show mean tensile residual stresses in the range of 28 MPa and 36 MPa being present in the core of the specimen. In the transition zone, the tensile residual stress level has a maximum value of 56 MPa (MV) and is therefore higher than that in the core. Near the martensitic rim, the residual stress level decreases again. Regarding the interpretation of the measurement results, it is important to point out that extracting the unit cell from the rebar specimen affects the residual stress state present in the unit cell. Hence, the residual stress states in the unit cell before and after the extraction differ. For the tangential stress component, however, they can be considered to be at least similar. This can also be seen in the numerical results presented in Section 4.2.2.

### 3.2. Microstructure Investigations

**Figure 5:**
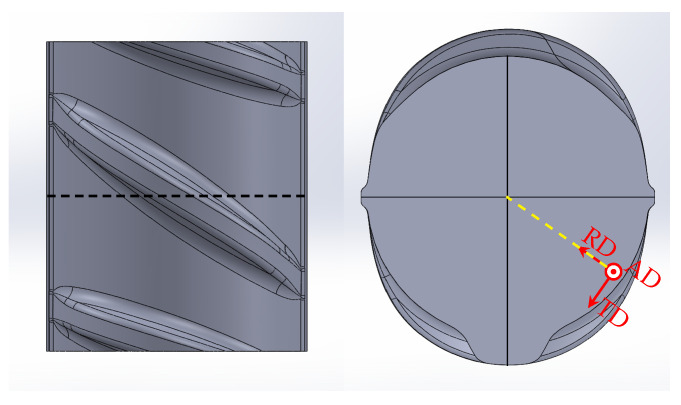
Idealized CAD representation of the rebar specimen used for the experimental analyses: the plane, in which the residual stress measurements in the core and the transition zone, the microstructure analysis, the hardness measurements and the EBSD measurements were conducted, is highlighted in black on the left side and displayed in sectional view on the right side. The path along which the microstructure analysis and the hardness measurements were carried out in the measurement plane is highlighted in yellow. The measurement location, at which the texture analysis was conducted, is highlighted in white. The local coordinate system of the measurement location is highlighted in red, whereas RD, AD and TD indicate the local radial, axial and tangential directions, respectively.

For microstructure analysis, the surface of the rebar specimen was ground and polished with the device Tegramin-30 from the manufacturer Struers (grinding in two steps with grade 320 and grade 1000 silicon carbide foils, polishing in two steps with two different diamond sprays; grain size of diamonds 3 µm and 1 µm). Then, the surface was etched with alcoholic nitric acid (HNO_3_, 2%). Micrographs of the microstructure were taken with the light microscope Aristomet from the manufacturer Leica. The path along which the micrographs were taken is highlighted in yellow in Figure 5 (right).

**Figure 6:**
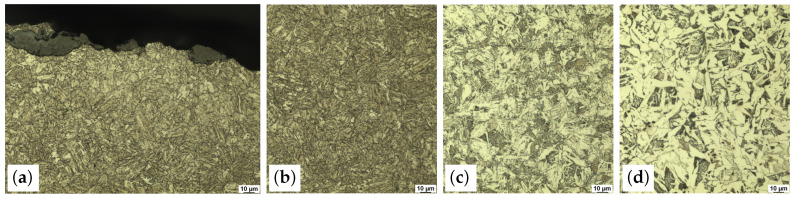
Micrographs at different locations of the cross-section of the rebar specimen: (**a**) thin surface layer directly beneath the rebar surface with differing microstructure, (**b**) rim with martensitic microstructure, (**c**) transition zone with bainitic microstructure and (**d**) core with ferritic–pearlitic microstructure.

The micrographs show a ferritic–pearlitic microstructure in the core of the specimen. In the transition zone and in the outer rim, a bainitic and a martensitic microstructure, respectively, can be observed (Figure 6). These observations are in accordance with the literature results [7,11]. The analysis of the microstructure also shows an oxide layer on the rebar surface. The oxide layer is disrupted at the investigated location. Its maximum thickness is about 20 µm. Underneath the oxide layer, another thin layer, whose microstructure differs from the microstructure in the martensitic rim, can be seen. With a thickness of about 50 µm, this second layer is significantly thicker than the oxide layer. The microstructure and the investigations of Volkwein et al. [7] indicate that this second layer is decarburized.

### 3.3. Hardness Measurements

**Figure 7:**
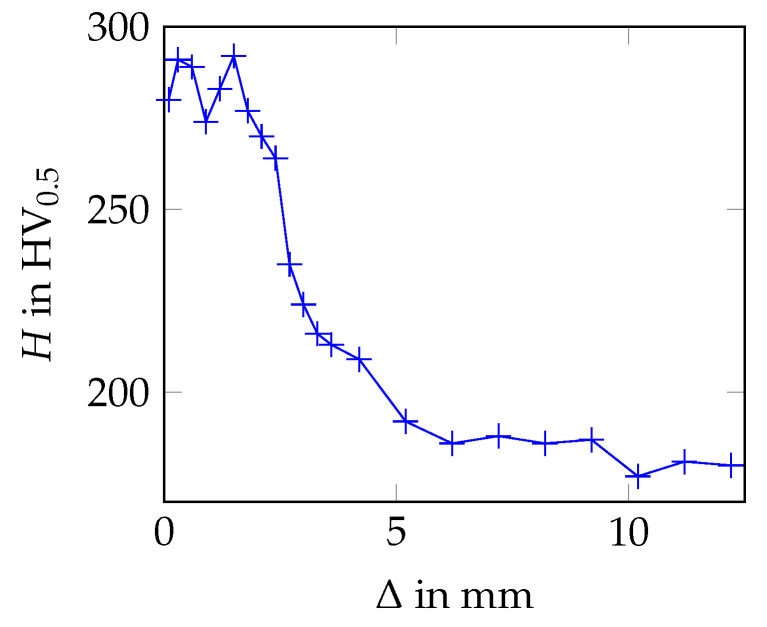
Hardness, *H*, as a function of the depth, Δ, (measured from the rebar surface) evaluated along the path highlighted yellow in Figure 5 (right).

For the hardness measurements, the specimen surface was prepared in the same way as for the microstructural analysis. Micro-hardness was measured using the hardness tester Qness 60 A+ with a load of 500 g and a dwell time of 15 s. The micro-hardness profile of the examined rebar specimen is shown in Figure 7. The profile was determined along the path shown in Figure 5 (right).

The measured hardness values are the highest in the martensitic rim, with a maximum value of 292 HV. At a depth of about 2.5 mm, a steep decrease of the hardness values occurs. In the core of the rebar specimen, the hardness is approximately 180 HV. The measured hardness values are in accordance with the literature results [7,11].

Based on the hardness profile, the thickness of the martensitic rim can be estimated to be about 2.5 mm, which also seems to be the case for the bainitic transition zone. Taking the actual diameter of the base cylinder of 26.80 mm into account and assuming an almost constant martensite fraction of $z_{\mathrm{mar}}\approx 1$ in the rim, the maximum martensite area fraction within the cross-section of the rebar specimen is approximately 34%. Making the same assumption for the bainitic transition zone ($z_{\mathrm{bai}}\approx 1$), the maximum bainite area fraction is approximately 27%. Hence, the area fractions of martensite, bainite and ferrite+pearlite are roughly the same within the cross-section of the investigated rebar specimen.

### 3.4. Texture Investigations

In order to investigate the crystallographic texture in the martensitic rim of the rebar specimen, EBSD measurements were carried out. The measurement plane and the exact measurement location are shown in Figure 5.

**Figure 8:**
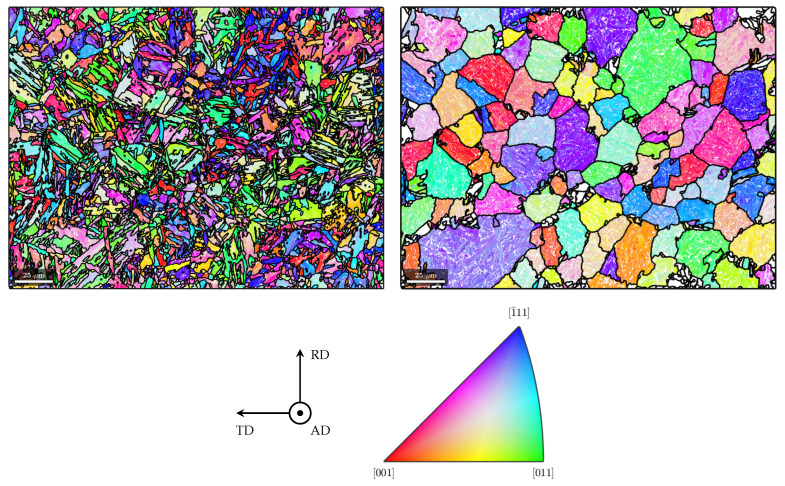
Inverse pole figure maps (AD||[hkl]) of martensite (**left**) and the reconstructed austenitic phase (**right**) [24,25].

**Figure 9:**
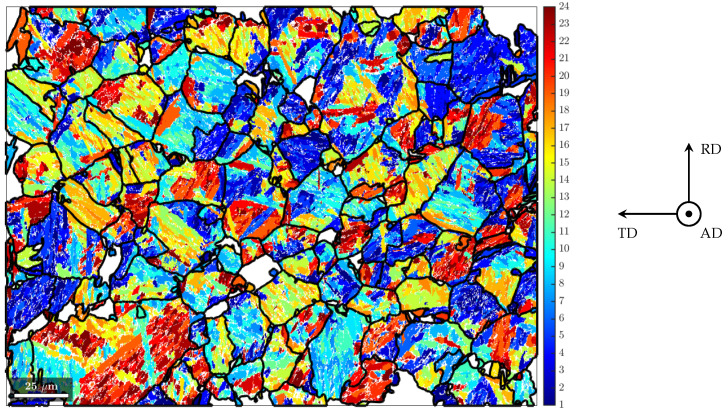
Results of austenite parent grain reconstruction, i.e., grain boundaries of reconstructed prior austenite grains, and results of the martensite variants analysis, i.e., individual martensite variants present in the prior austenite grains [24,25].

**Figure 10:**
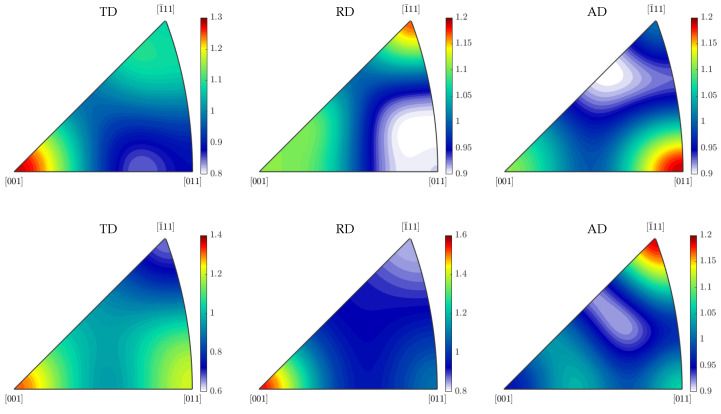
Inverse pole figures for the martensite phase (**top** row) and the austenite phase (**bottom** row) showing the inverse pole density function evaluated in the tangential, radial and axial directions of the specimen, respectively (see Figure 5 and Figure 8) [24,25].

For the EBSD measurements, the specimen surface was prepared in the same way as for the microstructural analysis and the hardness measurements. Additionally, extra-fine polishing in one step with fumed silica suspension was carried out (grain size of the silica oxide, 0.25 µm). The EBSD measurements were performed with the field emission scanning electron microscope JSM-7600F from the manufacturer JEOL (Tokyo, Japan) (distance between individual scanning points: 0.3 µm; accelerating voltage: 20 kV; working distance: 20 mm; tilt angle of measurement plane: 70°). Measurement results were processed with the Matlab toolbox MTEX [24,25,26] and are shown in Figure 8 (left). With the MTEX toolbox, both the prior austenite grains were reconstructed (Figure 8 (right)) and a martensite variant analysis was carried out using the Kurdjumov–Sachs orientation relationship (Figure 9). Furthermore, the inverse pole density functions were determined for both the martensite and the prior austenite (Figure 10).

The martensitic microstructure exhibits a weak crystallographic anisotropy (Figure 10, top row). From the results of the austenite parent grain reconstruction, mostly polygonal grains can be reported (Figure 8 (right)). The reconstructed prior austenite shows a weak crystallographic texture as well (Figure 10, bottom row). Only in some of the prior austenite grains, martensite variants with a preferred crystallographic orientation form (Figure 9).

## 4. Numerical Investigations

Within the scope of the numerical investigations, numerical simulations of the TempCore^TM^ process (CRM Group, Liège, Belgium) were carried out. As the extended modeling approach goes beyond the current state of research, a detailed explanation of the proposed methodology is given. The results of the numerical simulations were compared to the experimental results from Section 3 and from the literature.

### 4.1. Modeling

The numerical simulations of the TempCore^TM^ process were carried out using the finite element code Abaqus [27]. In the FE model, the heat-treatment was considered consisting of two subsequent steps, i.e., *quenching* and *air-cooling*. To solve the heat transfer problem and the mechanical problem, a sequential approach was chosen. Both problems were solved for different idealized periodic 2D- and 3D-model geometries.

#### 4.1.1. Geometry and Boundary Conditions

**Figure 11:**
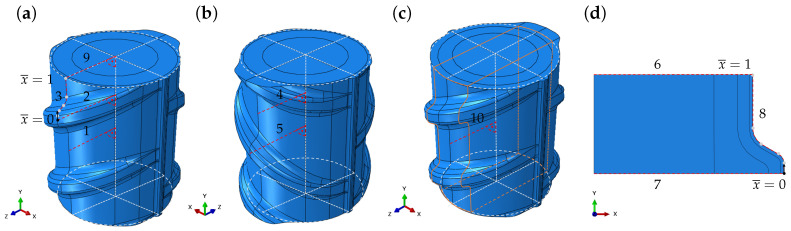
Different types of idealized model geometries, including specific evaluation paths: (**a**,**b**) *periodic unit cell* (rebar sides with alternating, viz., parallel ribs), (**c**) *periodic unit cell* with middle segment (highlighted in orange) and outer segments and (**d**) *axisymmetric geometry*. In subfigures (**a**,**d**), the different segments of path 3 and 8 are separated by gray dots. For paths 1, 2, 3, 4, 5, 9 and 10, the axial and tangential directions correspond to the global *y*- and *x*-directions, respectively. For paths 6, 7 and 8, the axial and tangential directions correspond to the global *y*- and *z*-directions.

In the model, the actual rebar geometry was idealized. The different types of idealized model geometries, which have been used (*periodic unit cell* and *axisymmetric geometry*), are shown in Figure 11. To generate the model geometries, the 3D-CAD program SOLID WORKS (2019) was used [28]. The model geometries were parameterized according to the specifications given in Table 3 (see also Figure 1). To discretize the *periodic unit cell*, DC3D4 and C3D4 elements were used in case of the thermal and mechanical problems, respectively. To discretize the *axisymmetric geometry*, DCAX4 and CAX4 elements were used [27].

Regarding the heat transfer problem, a large heat transfer coefficient, $h_{1}$, was applied on the lateral surface of the *periodic unit cell*, viz., the corresponding boundary of the *axisymmetric geometry* for a short period of time, $0<t\leq t_{1}$, during the *quenching* step. During the *air-cooling* step, the rebar was cooled to ambient temperature, $T_{\infty }$. The heat transfer coefficient, $h_{2}$, was small and the cooling time, $t_{1}<t\leq t_{2}$, was large [3]. The parameters for the heat transfer problem, including the initial temperature of the rebar, $T_{0}$, are given in Table 4. The parameter values used for this study were chosen based on the literature data. To ensure congruent temperature and displacement fields, appropriate (periodic) boundary conditions on the top and bottom surfaces of the *periodic unit cell* and the *axisymmetric geometry* were applied in cases of the thermal and mechanical problems. Regarding the mechanical problem, rigid body motions were suppressed by appropriate displacement boundary conditions.

In order to compare the numerical results of the simulations of the TempCore^TM^ process to the results of the residual stress measurements on the extracted unit cell from this work (see Section 3.1.2) and on the separated middle segment from the work of Volkwein et al. [7] (see Section 2.3 and Figure 11c), the stress redistribution as a consequence of the relaxation of the unit cell, viz., the middle segment due to extracting, viz., separating, had to be simulated. The relaxation of the unit cell was simulated in an additional numerical relaxation step, in which the periodic boundary conditions were released. The relaxation of the middle segment was simulated also in an additional numerical step, in which the material stiffness of the outer segments decreased linearly over the step time from its initial value to 1 Pa.

#### 4.1.2. Material Behavior of the Rebar Steel Grade B500B

To describe the thermo-mechanical material behavior of the rebar steel grade B500B, the approach suggested by Robl et al. [3] was extended. They considered the thermal material behavior of the rebar steel grade B500B as isotropic. The authors defined the thermal energy as a function of the specific heat capacity. To describe heat conduction, Fourier’s law was applied [27]. The mechanical material behavior was considered as isotropic and thermoelastic-idealplastic. The von Mises yield criterion and the associated flow rule were used to describe the plastic material behavior [30]. The kinetics of the phase transformation of austenite to martensite was captured using the Koistinen–Marburger model [31]. The kinetics of the formation of the other product phases, i.e., bainite, pearlite and ferrite, was captured using the JMAK model [32,33,34,35], whereby the mixture of these phases was considered as one single product phase. The volume change resulting from the transformation of austenite into each of the product phases was considered as well. The thermal material parameters, i.e., specific heat capacity and thermal conductivity, and the mechanical material parameters, i.e., Young’s modulus, Poisson’s ratio, linear thermal expansion coefficient and yield stress, were determined according to the local phase fractions using the linear rule of mixture.

The extension of the model includes the phenomenon of TRIP. Tempering of the martensitic rim is also considered in the extended model. For the latter purpose, the tempering kinetics of martensite in general and the kinetics of cementite precipitation in particular had to be depicted in the model. Furthermore, the change of the specific volume and the change of the thermo-mechanical material behavior due to tempering had to be addressed. To describe the thermo-mechanical material behavior, the user subroutines UMATHT and UMAT were used [27]. The material parameters used for the extended modeling approach were taken from the work of Robl et al. [3]. However, individual parameter values were adapted.

##### General Kinetics of Martensite Tempering

**Figure 12:**
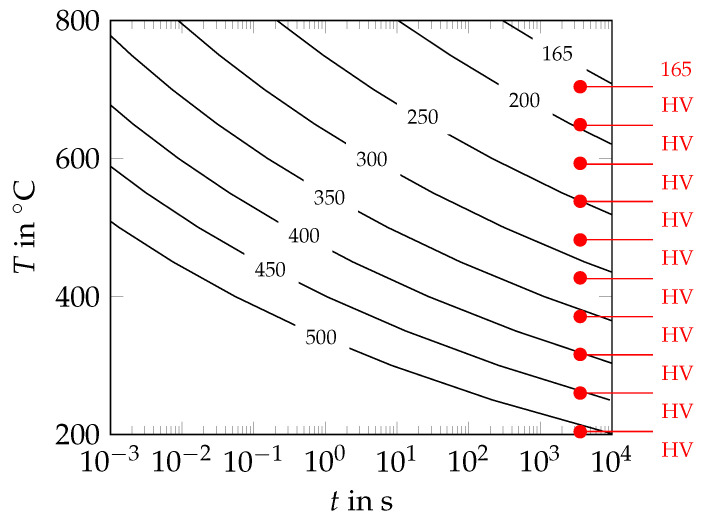
Martensite hardness (HV) as a function of the tempering time, *t*, and the tempering temperature, *T*, for the rebar steel grade B500B according to the model of Kang et al. [37] (isothermal temperature control, experimentally determined hardness values for 0.2 wt.% carbon steel with 0.8 wt.% Mn after tempering for 3600 s at different tempering temperatures highlighted in red [38]).

To describe the kinetics of martensite tempering in the model, the tempering ratio,

(1) $$R_{\mathrm{tem}}=\frac{H_{\mathrm{tmar}}-H_{\mathrm{mar}(0)}}{H_{\mathrm{mar}(\infty )}-H_{\mathrm{mar}(0)}},$$

was used. The tempering ratio was defined in terms of the Vickers hardness of untempered, (partly) tempered and fully tempered martensite, $H_{\mathrm{mar}(0)}$, $H_{\mathrm{tmar}}$, $H_{\mathrm{mar}(\infty )}$ [36]. If $R_{\;}\mathrm{tem}=0$ applied, no tempering had occurred yet. For a tempering ratio of $R_{\;}\mathrm{tem}=1$, the martensite was fully tempered. The hardness of (partly) tempered martensite,

(2) $$H_{\mathrm{tmar}}=\left(1542.97-\frac{25.31}{X_{C}}\right)\cdot \exp\left(-1.23\times 10^{-4}\times TP\right),$$

was determined with respect to the tempering parameter

(3) $$TP=T\cdot \left[\log\left(t\right)+\left(k_{0}+\sum_{j}k_{j}\cdot X_{j}\right)\right],$$

and the carbon content of a steel grade, $X_{C}$ [37]. The tempering parameter was given with respect to the tempering time, *t*; the tempering temperature, *T*; and the chemical composition of the steel grade, i.e., the amount of alloying elements, $X_{j}$. The parameter values for the coefficients of the alloying elements, $k_{j}$, as well as for the constant $k_{0}$, were determined by Kang et al. [37] for a wide range of low-alloyed steels. In our model, the hardness values were restricted to the range from 165 HV to 500 HV. These hardness values correspond to the hardness values of fully tempered and untempered martensite, respectively [11,38]. The isothermal time–temperature–hardness diagram according to Equations (2) and (3) is shown in Figure 12, from which it can be seen that the values for the martensite hardness predicted by the model are in reasonable accordance with the hardness measurements carried out by Grange et al. [38].

##### Kinetics of Cementite Precipitation

To describe the kinetics of cementite precipitation, the cementite precipitation ratio,

(4) $$R_{\mathrm{pcpt}}=\frac{500\;\mathrm{HV}-H_{\mathrm{tmar}}}{500\;\mathrm{HV}-355\;\mathrm{HV}},$$

was applied in the model, whereas $R_{\mathrm{pcpt}}=1$ applies for $H_{\mathrm{tmar}}\leq 355$ HV in general. The hardness range from 500 HV to 355 HV, in which the precipitation of cementite took place in the model, was specified as follows. For the steel grade C18, which has a similar chemical composition as the rebar steel grade B500B, the precipitation of cementite goes along with a total hardness decrease of 135 HV (from 470 HV to 335 HV) [13]. This is equivalent to a relative hardness decrease of 29%. For the rebar steel grade B500B, a relative hardness decrease of 29% corresponds to a hardness decrease of 145 HV (from 500 HV to 355 HV).

**Figure 13:**
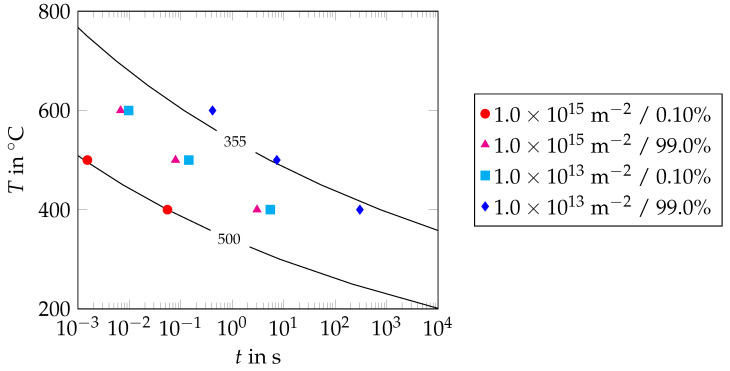
Time–temperature–precipitation (TTP) diagram of cementite in martensite for the rebar steel grade B500B, including the hardness curves for hardness values of 500 HV and 355 HV (hardness curves according to Equations (2) and (3)): transformation start (transformation ratio 0.001) and transformation end (transformation ratio 0.990) shown for two different dislocation densities ($1.0\times 10^{15}$ m${}^{-2}$, $1.0\times 10^{13}$ m${}^{-2}$).

To verify the modeling assumptions regarding precipitation start and end, isothermal precipitation kinetics simulations for the precipitation of cementite in martensite were undertaken using the software package MatCalc 6 [39,40]. For the simulations, the chemical composition of the rebar steel grade B500B according to Hameed et al. [11] was taken into account. The size of the martensite grains was determined as approximately 1.5 µm in terms of the equivalent radius, with the size of the prior austenite grains as approximately 7.5 µm [24,25]. The precipitation of cementite was assumed to take place at the boundaries of the prior austenite grains and the boundaries of the martensite laths [12]. A minimum nucleation radius of 0.337 nm was chosen. This value corresponds to half of the length of one unit cell of the cementite phase. To determine the start of cementite precipitation approximately, the simulations were carried out for a dislocation density of $1\times 10^{15}$ m${}^{-2}$, which corresponds to the dislocation density in as-quenched martensite [12,41]. To estimate the end of cementite precipitation as well, the simulations were carried out for a dislocation density of $1.0\times 10^{13}$ m${}^{-2}$ once again. The value used for the dislocation density in the latter case corresponds to the dislocation density in fully annealed martensite [40]. In Figure 13, the precipitation start (precipitation ratio 0.001) and the precipitation end (precipitation ratio 0.990) predicted by the MatCalc simulations are shown for both dislocation densities. Additionally, the hardness curves for hardness values of 500 HV and 355 HV are shown. The results in Figure 13 show that the precipitation start for a dislocation density of $1.0\times 10^{15}$ m${}^{-2}$ predicted by MatCalc approximately coincides with the 500 HV hardness curve. This also accounts for the precipitation end for a dislocation density of $1.0\times 10^{13}$ m${}^{-2}$ and the 355 HV hardness curve. Therefore, it can be concluded that the modeling assumptions regarding precipitation start and precipitation end are reasonable.

##### Change in the Specific Volume Due to Tempering

The change in the specific volume of martensite due to tempering,

(5) $$\frac{\Delta V}{V}=z_{\mathrm{mar}}\cdot R_{\mathrm{pcpt}}\cdot (\frac{\Delta V}{V}{)}_{\mathrm{tot}},$$

was modeled with respect to the cementite precipitation ratio. Its maximum value,

(6) $${\left(\Delta V/V\right)}_{\mathrm{tot}}=-{\left(\Delta V/V\right)}_{1}-{\left(\Delta V/V\right)}_{2}=-0.48\;\mathrm{vol.}\%,$$

only occurred for fully martensitic microstructures, i.e., $z_{\mathrm{mar}}=1$, and if the precipitation of cementite was completed, i.e., $R_{\mathrm{pcpt}}=1$. During the TempCore^TM^ process, this change in the specific volume is evoked by the diffusion of carbon atoms to dislocations and grain boundaries as well as by the precipitation of cementite. In the model, the decrease in the specific volume, which is associated with the diffusion of carbon atoms to dislocations and grain boundaries in the real process, was set to ${\left(\Delta V/V\right)}_{\;1}=0.15$ vol.% [42]. The decrease in the specific volume, which is associated to the precipitation of cementite in the real process,

(7) $$\begin{array}{cc} {\left(\Delta V/V\right)}_{2} & =\frac{\left(100-4\cdot X_{C}\right)\cdot \left(V_{\mathrm{fer}}/n_{\mathrm{fer}}\right)}{\left(100-X_{C}\right)\cdot \left(V_{\mathrm{mar}}/n_{\mathrm{fer}}\right)}\\ \; & +\frac{3\cdot X_{C}\cdot \left(V_{\mathrm{cem}}/n_{\mathrm{cem}}\right)-\left(100-X_{C}\right)\cdot \left(V_{\mathrm{mar}}/n_{\mathrm{mar}}\right)}{\left(100-X_{C}\right)\cdot \left(V_{\mathrm{mar}}/n_{\mathrm{fer}}\right)}, \end{array}$$

was set to 0.33 vol.% for $X_{C}=0.19$ wt.%. In Equation (7), the relative volumina of the unit cells of ferrite, martensite and cementite, $V_{\mathrm{fer}}$, $V_{\mathrm{mar}}$, $V_{\mathrm{cem}}$, were taken into account, as well as the numbers of iron atoms per unit cell, $n_{\mathrm{fer}}$, $n_{\mathrm{mar}}$, $n_{\mathrm{cem}}$ [43].

##### Changes in the Thermo-Mechanical Material Parameters

Robl et al. [3] considered the thermal material parameters, i.e., specific heat capacity and thermal conductivity, of all product phases, i.e., martensite, bainite, pearlite and ferrite, to be the same. This was also assumed for the elastic material parameters, i.e., Young’s modulus and Poisson’s ratio [11]. For this reason, the influence of tempering on the thermal and elastic material parameters was not considered in the extended model. However, the influence of tempering on the linear thermal expansion coefficient and the yield stress of martensite was taken into account. The linear thermal expansion coefficient of (partly) tempered martensite,

(8) $$\alpha_{\mathrm{tmar}}=\alpha_{\mathrm{mar}(0)}-\left[\alpha_{\mathrm{mar}(0)}-\alpha_{\mathrm{bpf}}\right]\cdot R_{\;}\mathrm{pcpt},$$

depends on the linear thermal expansion coefficients of bainite, pearlite, ferrite and untempered martensite, $\alpha_{\mathrm{bpf}}$ and $\alpha_{\mathrm{mar}(0)}$. The yield stress of (partly) tempered martensite,

(9) $$Y_{\mathrm{tmar}}=Y_{\mathrm{mar}(0)}-\left[Y_{\mathrm{mar}(0)}-Y_{\mathrm{mar}(\infty )}\right]\cdot R_{\;}\mathrm{tem},$$

depends on the yield stresses of untempered and fully tempered martensite, $Y_{\mathrm{mar}(0)}$ and $Y_{\mathrm{mar}(\infty )}$. While the linear thermal expansion coefficient is defined as a function of the precipitation ratio, $R_{\mathrm{pcpt}}$, the yield stress of martensite is a function of the tempering ratio, $R_{\mathrm{tem}}$. This is due to the assumption that in the real process the linear thermal expansion coefficient of tempered martensite is influenced mainly by the precipitation of cementite, but only to a minimal extent by the recrystallization of the microstructure. The yield stress, on the other hand, is influenced by both effects.

Before cementite precipitation starts in the model, the linear thermal expansion coefficient of martensite is equal to the linear thermal expansion coefficient of untempered martensite. After cementite precipitation is completed, $\alpha_{\mathrm{tmar}}=\alpha_{\mathrm{bpf}}$ applies. This is reasonable, as the ferrite content of fully tempered martensite is high and the linear thermal expansion coefficient of cementite is similar to those of bainite, pearlite and ferrite [44]. Before tempering starts, the yield stress of martensite is equal to the yield stress of untempered martensite in the model. After tempering is completed, the martensite yield stress is equal to the yield stress of fully tempered martensite. The yield stress of untempered martensite, $Y_{\mathrm{mar}(0)}$, is known from the flow curves set for the rebar steel grade B500B, which is given in the literature [11]. To describe the yield stress for temperatures below 200 °C in the model, the proportional limit, $R_{p0.2}$, was chosen. Above 300 °C, the stress was chosen, at which the stress–strain curve deviates from Hooke’s line for the first time, which is approximately $0.4\cdot R_{p0.2}$. The proportional limit of fully tempered martensite at room temperature, $Y_{\mathrm{mar}(\infty )}$, was approximated as follows. According to DIN 50150 [45], the hardness of fully tempered martensite in the rim of B500B rebars, $H_{\mathrm{mar}(\infty )}=165$ HV, corresponds to a tensile strength of 530 MPa. As strain hardening is reported to be negligible for low-carbon boron steel for sufficiently large tempering ratios [46], the proportional limit of fully tempered martensite at room temperature was also taken as 530 MPa. For elevated temperatures, the yield stress of fully tempered martensite,

(10) $$Y_{\mathrm{mar}(\infty )}\left(T\right)=Y_{\mathrm{mar}(\infty )}\left(0\;{}^{\circ}C\right)\cdot \frac{Y_{\mathrm{bpf}}\left(T\right)}{Y_{\mathrm{bpf}}\left(0\;{}^{\circ}C\right)},$$

was assumed to decrease in the same way as the yield stresses of bainite, pearlite and ferrite, $Y_{\mathrm{bpf}}$ [3]. All parameter values for the yield stresses of untempered and fully tempered martensite are given in Table 5.

##### Transformation-Induced Plasticity

In the model, TRIP strain increments associated to the mostly diffusive phase transformation,

(11) $$\Delta \varepsilon_{ij}^{\mathrm{TP},\mathrm{bpf}}=3\cdot K_{\mathrm{bpf}}\cdot \left(1-z_{\mathrm{bpf}}-z_{\mathrm{mar}}\right)\cdot \Delta z_{\mathrm{bpf}}\cdot S_{ij},$$

and the martensitic phase transformation,

(12) $$\Delta \varepsilon_{ij}^{\mathrm{TP},\mathrm{mar}}=3\cdot K_{\mathrm{mar}}\cdot \left(1-z_{\mathrm{bpf}}-z_{\mathrm{mar}}\right)\cdot \Delta z_{\mathrm{mar}}\cdot S_{ij},$$

are governed both by the same type of equation [47]. The TRIP strain increments for martensite, bainite, pearlite and ferrite, $\Delta \varepsilon_{ij}^{\mathrm{TP},\mathrm{mar}}$ and $\Delta \varepsilon_{ij}^{\mathrm{TP},\mathrm{bpf}}$, are defined as a function of the deviatoric stress, $S_{ij}$, of the phase fractions, $z_{\mathrm{bpf}}$ and $z_{\mathrm{mar}}$, and of their changes, $\Delta z_{\mathrm{bpf}}$ and $\Delta z_{\mathrm{mar}}$. The strain increments also depend on the transformation plasticity coefficients of martensite, bainite, pearlite and ferrite, $K_{\mathrm{mar}}$ and $K_{\mathrm{bpf}}$.

In the literature, Equations (11) and (12) have been widely used to describe TRIP for diffusive and martensitic phase transformations [47,48,49,50,51,52]. However, as the Maggee effect is not depicted adequately by Equation (12), it is not suitable to describe TRIP with regard to martensitic phase transformations in general [15,53]. Equation (12) is only suitable to describe TRIP with regard to martensitic phase transformations if no significant Magee effect can be considered to be present. As shown in Section 3.4, this is the case in the martensitic rim of B500B rebars.

**Figure 14:**
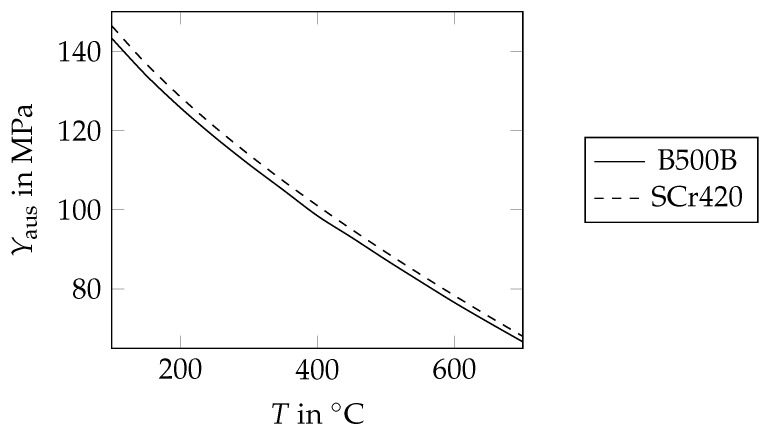
Yield stress of austenite, $Y_{\mathrm{aus}}$, as a function of the temperature, *T*, for the rebar steel grade B500B and the steel grade SCr420 according to the model of Eres–Castellanos et al. [54].

The parameter values for the transformation plasticity coefficients used in this study, $K_{\mathrm{mar}}=1.08\times 10^{-10}$ 1/Pa and $K_{\mathrm{bpf}}=1.40\times 10^{-10}$ 1/Pa, were determined experimentally by Otsuka et al. [47] for the steel grade SCr420 but not for the B500B rebar. Nevertheless, it seems permissible to use these parameter values also for the B500B rebar steel grade. This can be justified as follows. In general, the transformation plasticity coefficient is a function of the averaged transformation shear strain, the transformation volume strain and the yield strength of the matrix phase, i.e., austenite [15,16]. As the rebar steel grade B500B has a similar chemical composition compared to the steel grade SCr420 [11], the yield strengths of the austenite of both steels can be considered to be equal (Figure 14). This also applies to the transformation volume strain and the transformation shear strain [43].

##### Modified Material Parameters

Some of the parameter values used in this work to describe the material behavior of the rebar steel grade B500B differ from the values used in [3]:

- To describe the theoretical volume change at 0 °C resulting from the transformation of austenite to martensite, Robl et al. [3] used a value of ${\left(\Delta V/V\right)|}_{\mathrm{aus}\to \mathrm{mar}}=2.94$ vol.%. The authors determined this value by carrying out a dilatometer measurement with a cylindrical specimen, which was made from the rebar steel grade B500B. In the dilatometer measurement, the specimen was quenched from austenitic state to room temperature with a cooling rate of 215 K/s. This cooling rate, however, was significantly lower than the cooling rates occurring during the partial quenching step of the TempCore^TM^ process. Due to this lower cooling rate and the high martensite start temperature of the rebar steel grade B500B, it seems likely that diffusion of carbon atoms to dislocations and grain boundaries already occurred during measurement. Both effects are associated with a negative volume change of approximately 0.15 vol.% (see also Section 4.1.2) and most likely do not occur in the partial quenching step during the TempCore^TM^ process. For this reason, the value of the theoretical volume change resulting from the transformation of austenite to martensite at 0 °C had to be corrected to a value of 3.09 vol.%.
- To describe the theoretical volume change resulting from the transformation of austenite into the mixture of bainite, pearlite and ferrite at 0 °C, Robl et al. [3] determined a value of ${\left(\Delta V/V\right)|}_{\mathrm{aus}\to \mathrm{bpf}}=2.43$ vol.%. For the linear thermal expansion coefficient, the authors determined a value of $\alpha_{\mathrm{bpf}}=16.6\times 10^{-6}$ 1/K. Both values were identified by carrying out dilatometer measurements with a cylindrical specimen, which was cooled down from austenitic state to room temperature with a cooling rate of approximately 3 K/s. Due to the low cooling rate, however, the specimen exhibited a ferritic–pearlitic microstructure after cooling. Therefore, the values determined by the authors, ${\left(\Delta V/V\right)|}_{\mathrm{aus}\to \mathrm{pf}}=2.43$ vol.% and $\alpha_{\mathrm{pf}}=16.6\times 10^{-6}$ 1/K, corresponded to the transformation of austenite into the mixture of ferrite and pearlite. Hence, the volume expansion due to the transformation of austenite into the mixture of bainite, pearlite and ferrite during the TempCore^TM^ process as well as the linear thermal expansion coefficient of this mixture, were captured only approximately in the model of Robl et al. [3]. An improved approximation of both parameter values is provided by the following approach. For low-alloyed steels, the theoretical volume change resulting from the transformation of austenite into bainite at 0 °C has been reported as approximately ${\left(\Delta V/V\right)|}_{\mathrm{aus}\to b}{=\left(\Delta V/V\right)|}_{\mathrm{aus}\to \mathrm{mar}}-{\left[\left(\Delta V/V\right)\right|}_{\mathrm{aus}\to \mathrm{mar}}-\left(\Delta V/V\right){|}_{\mathrm{aus}\to \mathrm{pf}}]/2$ in the literature [55]. The linear thermal expansion coefficient has been reported as approximately $\alpha_{b}=\alpha_{\mathrm{mar}}+\left(\alpha_{\mathrm{pf}}-\alpha_{\mathrm{mar}}\right)/2$ [55]. Taking this into account, the theoretical volume change due to the transformation of austenite into the mixture of bainite, pearlite and ferrite, ${\left(\Delta V/V\right)|}_{\mathrm{aus}\to \mathrm{bpf}}=2.61$ vol.%, and the linear thermal expansion coefficient of this mixture, $\alpha_{\mathrm{bpf}}=15.9\times 10^{-6}$ 1/K, was approximated by using the linear rule of mixture. For this, the area fractions of bainite and of pearlite+ferrite in the cross-section of the rebar specimen estimated in Section 3.3 were taken into account.

#### 4.1.3. Material Behavior of the Thin Surface Layer

In Section 3.2, a thin surface layer with an altered microstructure was observed for the examined rebar specimen. To investigate numerically if this surface layer may be the reason for the high near-surface stress gradients observed by Hameed et al. [11], the material behavior of this layer had to be depicted in the model. This was performed in a simplified manner. Because the oxide layer at the surface is disrupted and the thickness of the oxide layer is small compared to the decarburized layer beneath, only the material behavior of the decarburized layer was depicted in the model. Furthermore, the assumption was made, that the layer is almost fully decarburized over its whole thickness. This assumption implies that the microstructure of the layer can be considered as almost fully ferritic after short tempering times.

In the model, the thermal material behavior of the thin surface layer was considered to be the same as the thermal material behavior of the martensite phase of the steel grade B500B. Due to the low thickness of the surface layer and its little influence on the heat transfer problem, this simplification therefore seems permissible. The mechanical material behavior of the thin surface layer was considered as isotropic and thermoelastic-idealplastic. To describe the plastic material behavior, the associated flow rule and the von Mises yield criterion were used.

All of the austenite within the thin surface layer was assumed to transform into martensite within the temperature range of 450 °C to 300 °C during the *quenching* step. To some extent, the upper bound of this temperature range has been freely chosen. However, it is above the martensite start temperature of 420 °C of the steel grade B500B. Furthermore, it is below the martensite start temperature of the thin surface layer, which is 476 °C according to the formula of Liu et al. [56]. For the calculation, the carbon content in the surface layer was assumed to be only 0.02 wt.%. The lower end of the temperature range corresponds to the temperature at which the martensitic transformation for the steel grade B500B is completed to an extent of 90% [3].

In the model, the thin surface layer exhibited the same elastic parameters, $E_{\mathrm{sl}}$ and $\nu_{\mathrm{sl}}$, as the rebar steel grade B500B [3]. The thermo-metallurgical eigenstrains that the thin surface layer was subjected to during cooling were also modeled in a simplified manner. During the *quenching* step, the linear thermal expansion coefficient of the austenite of the rebar steel grade B500B, $\alpha_{\mathrm{aus}}=23.4\times 10^{-6}$ 1/K, was assigned to the surface layer at temperatures above 450 °C. Below 300 °C, the linear thermal expansion coefficient of the mixture of pearlite and ferrite of the rebar steel grade B500B, $\alpha_{\mathrm{sl}}=\alpha_{\mathrm{pf}}=16.6\times 10^{-6}$ 1/K, was assigned to the thin surface layer (see Section 4.1.2). In the temperature range of 300 °C to 450 °C, the combined linear thermal expansion coefficient, $\alpha_{\mathrm{sl}}=-6.07\times 10^{-6}$ 1/K, by which also the volume expansion due to the transformation of austenite to martensite has been taken into account, was assigned to the thin surface layer. During the *air-cooling* step, the surface layer exhibited the linear thermal expansion coefficient of the mixture of pearlite and ferrite of the rebar steel grade B500B again, but over the whole temperature range. During the *quenching* step, the thin surface layer exhibited the yield stress of the austenite of the rebar steel grade B500B, i.e., $Y_{\mathrm{sl}}=Y_{\mathrm{aus}}$, above 450 °C, and below 300 °C, it exhibited the yield stress of the mixture of bainite, pearlite and ferrite, i.e., $Y_{\mathrm{sl}}=Y_{\mathrm{bpf}}$. Between 300 °C and 450 °C, the yield stress was linearly interpolated. During the *air-cooling* step, the yield stress was that of the mixture of bainite, pearlite and ferrite again, i.e., $Y_{\mathrm{sl}}=Y_{\mathrm{bpf}}$.

### 4.2. Results and Discussion

#### 4.2.1. Phase Distribution and Mechanical Properties

**Figure 15:**
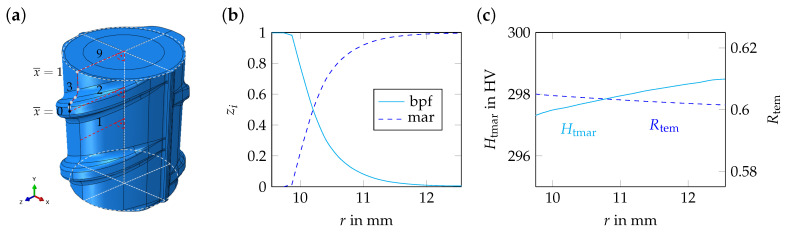
Numerical results for path 1, i.e., for (**a**) the *periodic unit cell*, at t=3000 s: (**b**) phase fractions of martensite, bainite, pearlite and ferrite, $z_{\mathrm{mar}}$, $z_{\mathrm{bpf}}$, as well as (**c**) martensite hardness, $H_{\mathrm{tmar}}$, and tempering ratio, $R_{\mathrm{tem}}$, as a function of the radius, *r*.

In Figure 15b, the phase fractions of martensite, bainite, pearlite and ferrite, $z_{\mathrm{mar}}$ and $z_{\mathrm{bpf}}$, are depicted for path 1 (*periodic unit cell*, see Figure 15a). In Figure 15c, the martensite hardness, $H_{\mathrm{tmar}}$, and the tempering ratio, $R_{\mathrm{tem}}$, are shown. The following observations can be made. In the center and the transition zone of the model, ferrite, pearlite and bainite are reported as the constituting phases. In the outer rim, mostly martensite is present. Up to a depth of 2.35 mm (measured from the rebar surface), the martensite fraction is larger than 0.50. At a depth of 2.70 mm, no martensite is formed anymore. The martensite hardness, $H_{\mathrm{tmar}}=298$ HV, is almost constant over the whole thickness of the outer rim. Also, the tempering ratio, $R_{\mathrm{tem}}=0.60$, is almost constant. Related to the cross-section area of the base cylinder, $A_{\mathrm{bar}}$, the martensite area fraction is 0.30. The area fraction of ferrite, pearlite and bainite is $z_{\mathrm{bpf},A}=1-z_{\mathrm{mar},A}=0.70$. Considering the tempering ratio, $R_{\mathrm{tem}}=0.60$, the martensite yield stress, $Y_{\mathrm{tmar}}$, is 844 MPa (Equation (10)). The yield stress of bainite, pearlite and ferrite is $Y_{\mathrm{bpf}}=431$ MPa. Using the linear rule of mixture, the yield stress of the *periodic unit cell* is approximately

(13) $$Y_{\mathrm{bar}}=Y_{\mathrm{tmar}}\cdot z_{\mathrm{mar},A}+Y_{\mathrm{bpf}}\cdot z_{\mathrm{bpf},A}=\left(844\times 0.30+431\times 0.70\right)\;\mathrm{MPa}=555\;\mathrm{MPa}.$$

These results are in good agreement with the experimental results from Section 3 and those of Rappl et al. [21]. In Section 3, a maximum martensite area fraction of 34% was determined. The thickness of the outer martensitic rim was approximately 2.5 mm, with the maximum hardness value of 292 HV. Rappl et al. [21] conducted tensile tests on rebar specimens with a nominal diameter of d=28 mm and determined the proportional limit of these specimens as $R_{p0.2}=574\pm 6$ MPa. This value is in good agreement with the calculated value for the yield stress (555 MPa).

#### 4.2.2. Residual Stress Distribution after Complete Cooling

**Figure 16:**
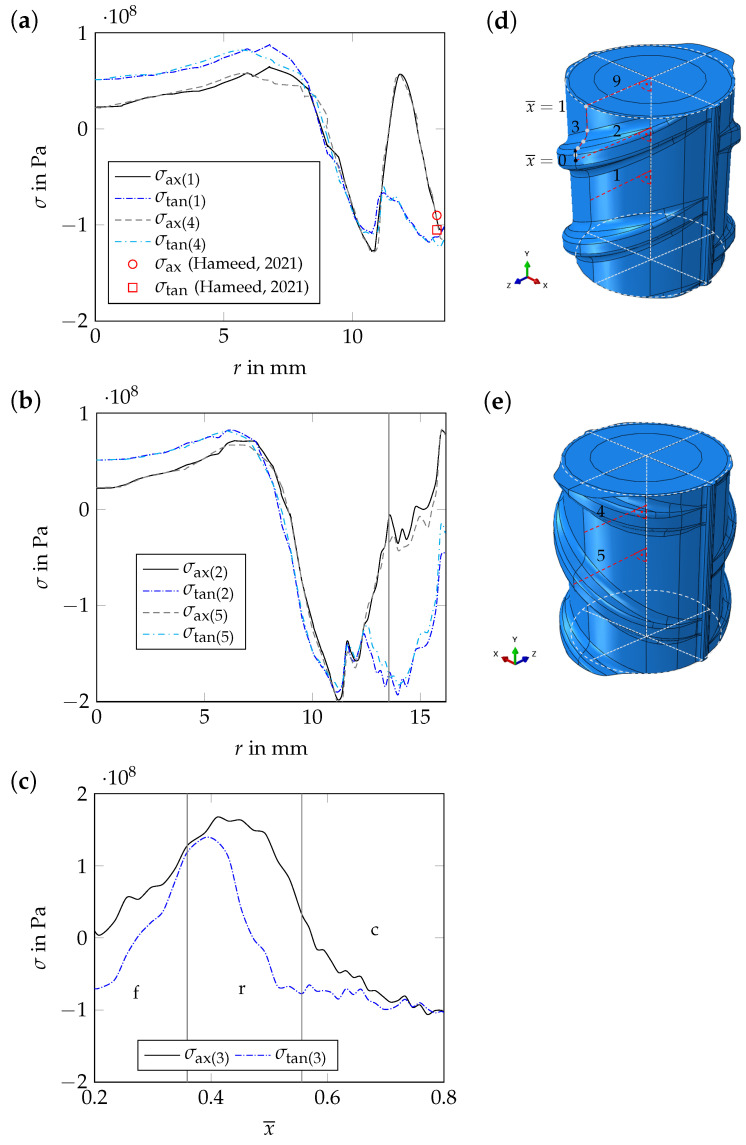
Numerical results for t=3000 s: (**a**–**c**) axial and tangential residual stress components, $\sigma_{\mathrm{ax}}$ and $\sigma_{\tan}$, as a function of the radius, *r*, and as a function of the normalized path variable, $\bar{x}$ (evaluation for paths 1, 2, 3, 4 and 5, i.e., for the *periodic unit cell*, see subfigures (**d**,**e**)). In subfigure (**a**), the results of the residual stress measurements of Hameed et al. [11] at a depth of 0.3 mm are given additionally (depth measured from the rebar surface, measurement location between two alternating ribs). In subfigure (**c**), the individual sections of path 3 (see Figure 11) are separated by vertical gray lines (c: outer surface of base cylinder; r: rib foot radius; f: rib flank).

Evaluating the resulting residual stress distribution after complete cooling shows tensile residual stresses in the core of the *periodic unit cell* in the axial and tangential directions (Figure 16a,b). In both directions, compressive residual stresses can be reported in the transition zone. In the martensitic rim, only compressive residual stresses occur in the tangential direction. In the axial direction, also tensile residual stresses can be reported beneath the surface (Figure 16a). On the model surface, compressive residual stresses occur between ribs (Figure 16a). At the tip of the ribs, tensile and compressive residual stresses are present in the axial and tangential directions, respectively (Figure 16b,c). The highest tensile residual stresses on the model surface occur in the rib foot radius regions (Figure 16c).

**Figure 17:**
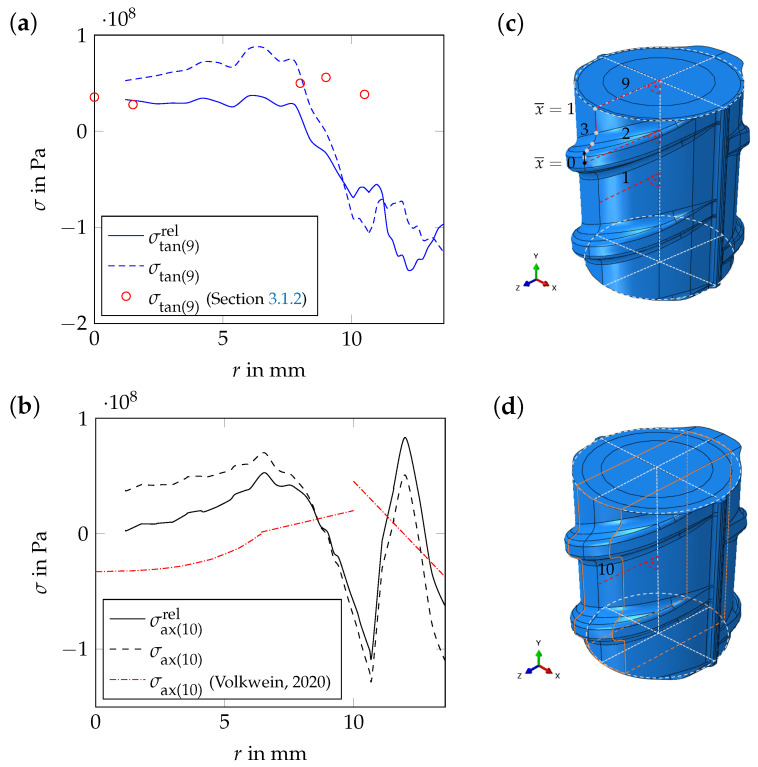
Numerical results: (**a**) tangential residual stress component, $\sigma_{\tan}$, as a function of the radius, *r*, (evaluation for path 9, i.e., for the *periodic unit cell*, for t=3000 s and after the relaxation step; see subfigure (**c**)) and (**b**) axial residual stress component, $\sigma_{\mathrm{ax}}$, as a function of the radius, *r*, (evaluation for path 10, i.e., for the *periodic unit cell*, for t=3000 s and after the relaxation step; see subfigure (**d**)). In subfigure (**a**), additionally the results from Section 3.1.2 are given (see also Figure 4 (right)), and in subfigure (**b**), the results of Volkwein et al. [7] are given (see also Section 2).

For path 1, which ends on the model surface between two ribs on the rib side with parallel ribs, and for path 4, which also ends on the rebar surface between two ribs, but on the rib side with alternating ribs, the results are similar. This applies also for paths 2 and 5, which both end at the tip of a rib. Hence, for the core and the transition zone, an axisymmetric stress state can be reported (Figure 16a,b). On the model surface, the stress state is not axisymmetric (Figure 16a–c). The evaluation of the residual stress distribution after the additional numerical relaxation steps, i.e., after releasing the periodic boundary conditions, viz., decreasing the material stiffness in the outer segments of the *periodic unit cell*, shows a significant stress redistribution in both cases. In Figure 17a, the tangential residual stress component after relaxation is shown for path 9. In Figure 17b, the axial stress component is shown for path 10.

On the model surface, the numerical results fit well with the experimental results of Hameed et al. [11] and Zheng et al. [8]. Between two ribs, Hameed et al. [11] determined axial compressive residual stresses of approx. −90 MPa and tangential compressive residual stresses of approx. −105 MPa at a depth of 0.3 mm (measured from the rebar surface, see Section 2.3 and Figure 16a). Zheng et al. [8] measured axial compressive residual stresses between −80 MPa and −90 MPa on the surface of rebar steels with diameters of d={16,24,32} mm. At the center, the numerical results fit well with the results of the residual stress measurements from this work (see Section 3.1.2 and Figure 17a). In the transition zone, however, the numerical results differ quantitatively from the experimental results. This may be due to the fact that bainite, which is the main microstructural constituent in the transition zone of the rebar specimen examined in Section 3, is not considered as an individual product phase in the model but as a part of the mixture of bainite, pearlite and ferrite. Therefore, TRIP might be depicted only approximately in this region of the model. The residual stress level predicted by the numerical simulation is in good agreement with the results of the residual stress measurements of Volkwein et al. [7] (see Section 2.3 and Figure 17b). The characteristic of the axial residual stress component in the core and in the martensitic rim as determined by Volkwein et al. [7] is in agreement with the numerical results, at least qualitatively. However, the compressive residual stress level in the core is underestimated by the model, while the compressive residual stress level on the rebar surface is overestimated. In the transition zone, the numerical results differ qualitatively and quantitatively from the measurement results. Again, not considering bainite as an individual microstructural constituent in the model could be the reason for these deviations. The deviations in the core and in the martensitic rim in contrast, may be due to the fact that the rebar specimen examined by Volkwein et al. [7]—even if it is from the same manufacturer—is from a different batch as the rebar specimen examined in Section 3, which results in different chemical compositions of both specimens.

#### 4.2.3. Effect of the Thin Surface Layer on Near-Surface Residual Stresses

**Figure 18:**
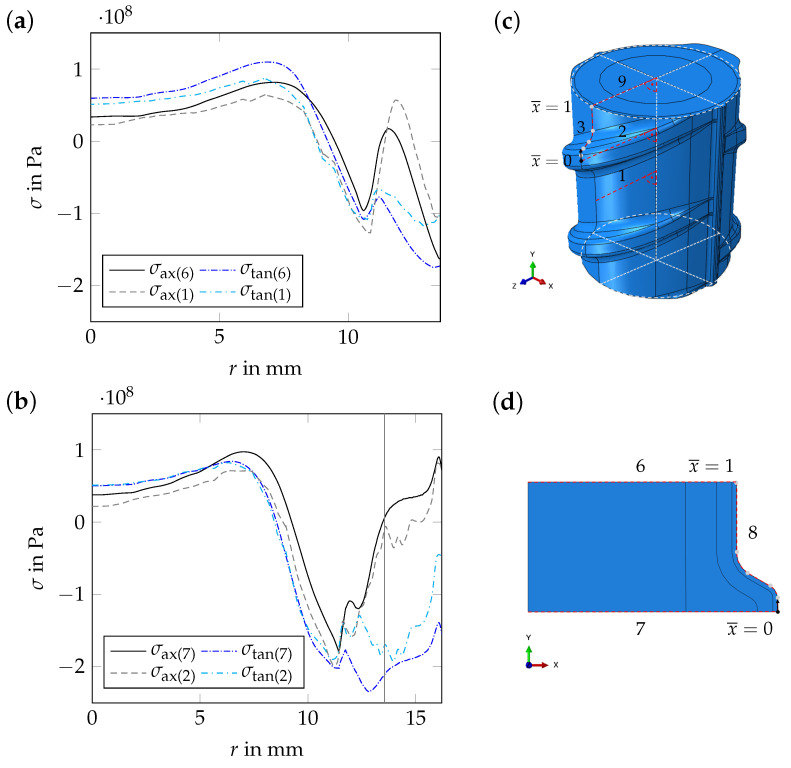
Numerical results for t=3000 s: (**a,b**) axial and tangential residual stress components, $\sigma_{\mathrm{ax}}$ and $\sigma_{\tan}$, as a function of the radius, *r*. Evaluation for paths 1 and 2, i.e., for the *periodic unit cell*, (see subfigure (**c**)) as well as for paths 6 and 7, i.e., for the *axisymmetric geometry* (see subfigure (**d**)).

The effect of the thin surface layer, which exhibits an altered microstructure, on the residual stress distribution in rebars, was investigated numerically using the *axisymmetric geometry*. The numerical results obtained using the *axisymmetric geometry* are similar to the results obtained using the *periodic unit cell* (Figure 18), but the computational costs are substantially lower in the first case. Quantitative deviations of the results mostly occur in the martensitic rim. For the *axisymmetric geometry*, both the axial and tangential residual stress components are shifted towards the compressive stress range. As the volume of the transverse ribs is larger for the *axisymmetric geometry* than for the *periodic unit cell*, these deviations are plausible (see Section 4.2.4).

**Figure 19:**
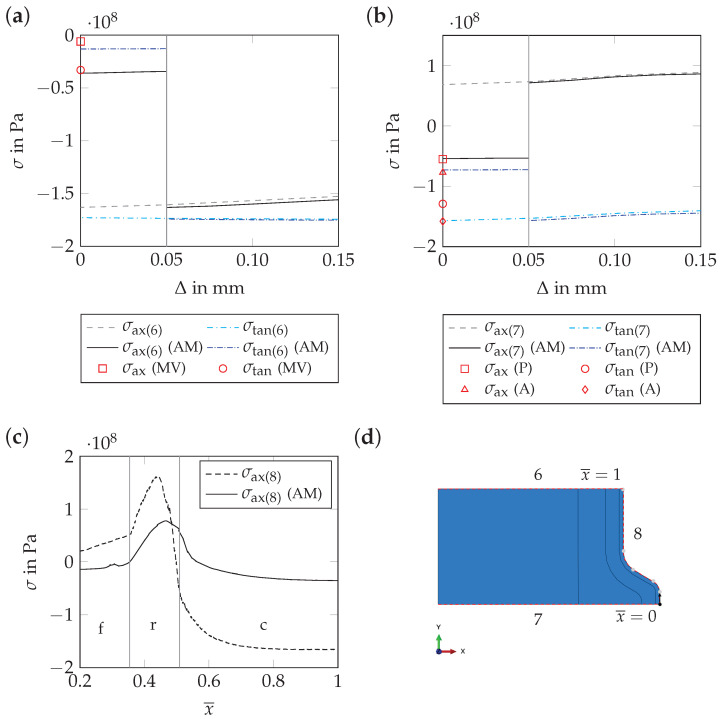
Numerical results for t=3000 s: axial and tangential residual stress components, $\sigma_{\mathrm{ax}}$ and $\sigma_{\tan}$, as a function of depth, Δ, (measured from the rebar surface), viz., the normalized path variable, $\bar{x}$, once neglecting and once considering the altered material behavior (AM) of the thin surface layer (evaluation for paths 6, 7 and 8, i.e., the *axisymmetric geometry*; see subfigure (**d**)). In subfigure (**c**), the individual sections of path 3 (see Figure 11d) are separated by vertical gray lines (c: outer surface of base cylinder; r: rib foot radius; f: rib flank). In subfigures (**a**,**b**), additionally, the results of the residual stress measurement from Section 3.1.1 are shown. The mean value determined in Section 3.1.1 for all measurements between parallel and alternating ribs is indicated as MV. The letters P and A indicate whether a specific measurement value has been determined on the rebar side with parallel or alternating ribs.

Considering the altered material behavior of the thin surface layer (thickness of 50 µm in the model), the residual stress distributions in the martensitic rim, the transition zone and the core of the model are barely influenced. Inside the thin surface layer, however, the residual stress level between two ribs decreases in the axial and tangential directions (Figure 19a). At the tip of the rib, the residual stress level in the tangential direction decreases as well. In the axial direction, the altered material behavior even leads to compressive instead of tensile residual stresses (Figure 19b). In the critical region of the rib foot radius, the altered material behavior leads to lower maximum tensile residual stresses (Figure 19c).

The numerical results for the thin surface layer are in reasonable accordance with the results of the residual stress measurements of Hameed et al. [11], which were carried out on the rebar surface. Regarding the residual stress measurements from Section 3.1.1, the numerical results are also in reasonable accordance. While the residual stress level on the rebar surface between two ribs is predicted qualitatively and quantitatively correct by the model, this only applies to the axial residual stress component at the tip of ribs. The tangential residual stress level predicted by the model is too low (Figure 19).

#### 4.2.4. Origin of the Residual Stresses

**Figure 20:**
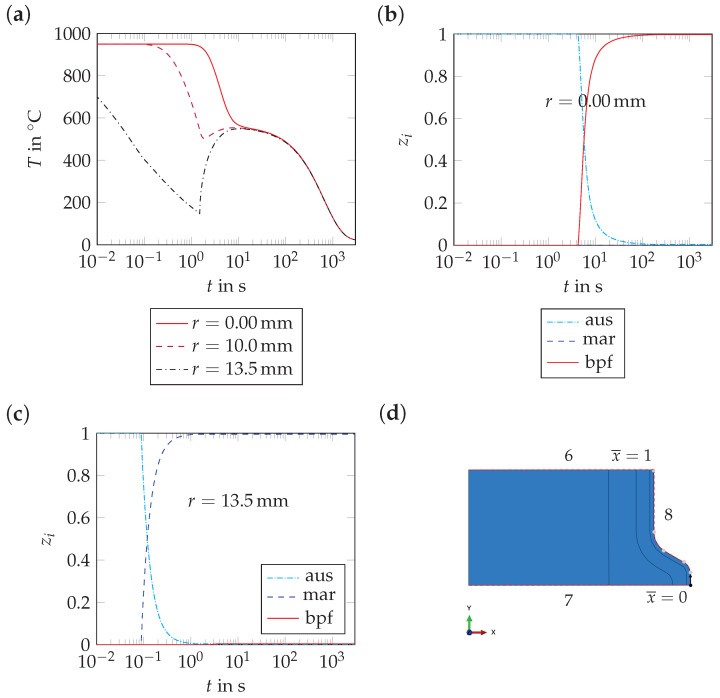
Numerical results: (**a**) evolution of temperature, *T*, and (**b**,**c**) evolutions of the phase fractions, $z_{i}$, at selected locations of path 6, i.e., for the *axisymmetric geometry* (see subfigure (**d**)).

**Figure 21:**
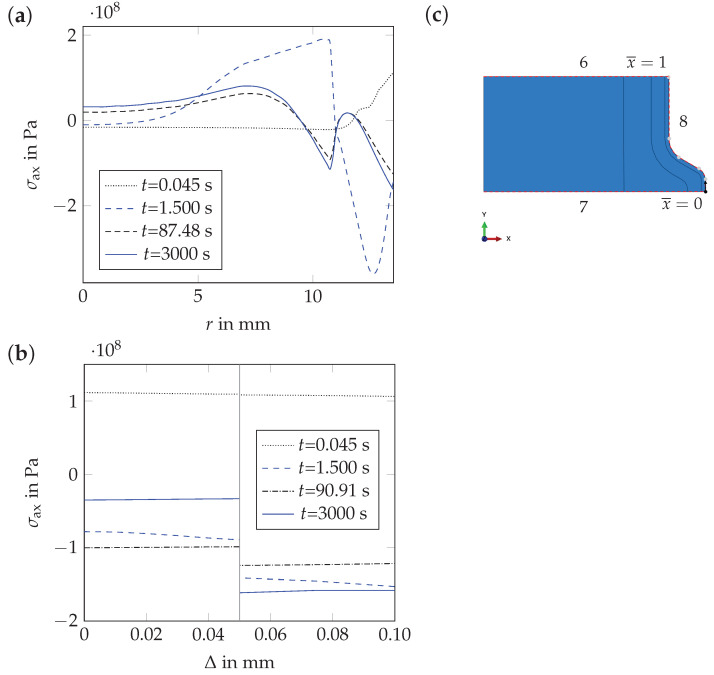
Numerical results for various cooling times, $t_{i}$: (**a**) axial stress component, $\sigma_{\mathrm{ax}}$, as a function of the radius, *r*, (evaluation for path 6, i.e., the *axisymmetric geometry*, altered material behavior of the thin surface layer not considered in the model), (**b**) axial stress component, $\sigma_{\mathrm{ax}}$, as a function of the depth, Δ, (depth measured from the rebar surface, evaluation for path 6, altered material behavior of the thin surface layer considered in the model) and (**c**) path locations.

The origin of residual stresses in rebars during cooling can be understood from the results depicted in Figure 20 and Figure 21. For t<0.045 s, lower temperatures in the rim and the transition zone than in the core of the model lead to larger shrinkage of the rim. Hence, tensile stresses in the rim and compressive stresses in the core develop. For 0.045s < t≤ 1.5 s, martensite formation takes place. At t=1.5 s, high compressive stresses are generated in the martensitic rim, as the transformation of austenite into martensite is associated with a volume expansion. The formation of the mixture of bainite, pearlite and ferrite ends after approximately 90 s. As the formation of this mixture is also associated with a volume expansion, the residual stresses at the model surface decrease again. The residual stress distribution changes only to a limited extent for t>90 s, as the linear thermal expansion coefficient of the tempered martensitic rim ($\alpha_{\mathrm{tmar}}=15.0\times 10^{-6}$ 1/K) is similar to those of the core and the transition zone ($\alpha_{\mathrm{bpf}}=15.9\times 10^{-6}$ 1/K).

Also in the thin surface layer, tensile stresses develop before the martensitic transformation starts. At the end of *quenching*, compressive stresses are predicted as well. The compressive stresses on the rebar surface are lower than in the martensitic rim because the volume expansion associated to the formation of martensite in the thin surface layer is smaller than in the martensitic rim. As the core and the transition zone transform from austenite into the mixture of bainite, pearlite and ferrite, the residual stress level decreases in the martensitic rim, but not significantly in the surface layer. For t>90 s, the compressive stress level decreases in the surface layer due to the fact that the linear thermal expansion coefficient of the surface layer ($\alpha_{\mathrm{sl}}=16.6\times 10^{-6}$ 1/K) is larger than that of the martensitic rim.

## 5. Summary

To extend the knowledge about residual stresses in rebars, residual stress measurements on a rebar specimen with a diameter of d=28 mm fabricated from the rebar steel grade B500B were carried out in this work. In addition, numerical simulations of the TempCore^TM^ process were undertaken.

The results of the residual stress measurements on the examined rebar specimen showed mean tangential tensile residual stresses in the range of 28 MPa to 36 MPa in the core and of 56 MPa in the transition zone. On the rebar surface, axial and tangential compressive residual stresses of −6 MPa, viz., −33 MPa were determined on average. At the tip of the ribs, high axial and tangential compressive residual stresses of up to −158 MPa were determined.

The results of the numerical investigations are in reasonable accordance with the experimental results from this work and from the literature. In the rib foot radius regions of the transverse ribs, the numerical results indicate moderate tensile residual stresses occurring at the rebar surface. High stress gradients directly beneath the rebar surface, which are reported in the literature and which are most likely related to a thin decarburized surface layer, could be reproduced qualitatively with the numerical model used.

To predict the residual stress level and the residual stress distribution in rebars in an at least qualitatively correct manner, not only the kinetics of the phase transformations during the TempCore^TM^ process and the volume changes, which are associated to the phase transformations, had to be depicted in the model. Also, TRIP and tempering of the martensitic rim during reheating had to be considered. To reproduce the high stress gradients directly beneath the rebar surface, the altered material behavior of the thin surface layer had to be depicted in the model.

Regarding the fatigue performance of B500B rebars, tensile residual stresses in the foot radius regions of transverse ribs as predicted by the model are highly noteworthy. In order to improve the fatigue performance of B500B rebars, it is of high interest how these stresses can be influenced on purpose, e.g., by varying individual process or geometry parameters. The clarification of this question is the focus of ongoing numerical investigations. For these investigations, the model geometry has to be adapted. For model geometries with diameters others than 28 mm, new sets of heat-treatment parameters have to be identified. An adaptation of the material model is not required. 

## Figures and Tables

**Table 1:** Results of the residual stress measurements on the rebar surface in MPa (for the measurement locations A–J see Figure 3).

Stress Component	Symbol	A	B	C	D	E	F	G	H	I	J
Axial stress component	$\sigma_{\mathrm{ax}}$	−2	−11	−8	−1	−22	−77	−9	−55	7	0
Tangential stress component	$\sigma_{\tan}$	−24	−41	−15	−36	−44	−158	−49	−129	−34	−20

**Table 2:** Results of the residual stress measurements (tangential stress component, $\sigma_{\tan}$, in MPa) in the core and the transition zone of the extracted unit cell (for the different measurement locations, see Figure 4 (left)): for each radius value, *r*, the mean value (MV) and the standard deviation (STD) of the respective measurement values were determined, whereas the measurement values in brackets were not considered because the micrographs of the associated measurement locations showed surface scratches.

*r* in mm	0°	90°	180°	270°	MV	STD
0.0	46.0	32.9	46.7	20.2	35.5	10.9
1.5	36.0	19.3	(−27.4)	(10.6)	27.7	8.4
8.0	(−35.8)	44.8	47.9	57.1	49.9	5.2
9.0	(8.2)	60.4	(67.9)	51.6	56.0	4.4
10.5	50.5	(46.9)	29.3	35.0	38.3	9.0

**Table 3:** Parameter set following DIN 488-2 [1] to specify the rebar model geometry.

Rebar	Symbol	Reference Value	Unit
Rebar diameter ^(1)^	*d*	28.00	mm
Bar diameter ^(2)^	$d_{\mathrm{bar}}$	27.10	mm
**Transverse Ribs**	**Symbol**	**Reference Value**	**Unit**
Distance between rib rows	*e*	2.00	mm
Rib spacing	*c*	16.80	mm
Foot radius	$r_{f}$	1.60	mm
Tip radius	$r_{t}$	0.84	mm
Rib width	*b*	2.80	mm
Rib heigth (middle of the rib)	$a_{m}$	2.60	mm
Rib heigth (one-/three-quarter point)	$a_{1/4}$, $a_{3/4}$	1.80	mm
Flank inclination	α	65	${}^{\circ }$
Rib inclination	β	60	${}^{\circ }$
**Longitudinal Ribs**	**Symbol**	**Reference Value**	**Unit**
Foot radius	$r_{f,l}$	1.00	mm
Tip radius	$r_{t,l}$	0.60	mm
Rib width	$b_{l}$	2.00	mm
Rib heigth	$a_{l}$	0.60	mm
Flank inclination	$\alpha_{l}$	90	${}^{\circ }$

^(1)^ Weight-specific diameter; ^(2)^ Diameter of the base cylinder, see DIN 488-2 [1].

**Table 4:** Parameters used in the model to describe the TempCore^TM^ process for rebars with a diameter of d=28 mm [29].

$T_{0}$ [${}^{\circ }$C]	$T_{\infty }$ [${}^{\circ }$C]	$t_{1}$ [s]	$t_{2}$ [s]	$h_{1}$ [W/m^2^K]	$h_{2}$ [W/m^2^K]
950 ^(1)^	20 ^(2)^	1.5 ^(3)^	2998.5 ^(4)^	34,000 ^(3)^	40 ^(1)^

^(1)^ Bandyopadhyay et al. [29] reported these values for both a rebar with a diameter of d=32 mm and a rebar with a diameter of d=16 mm. ^(2)^ This value corresponds to room temperature. ^(3)^ For a rebar with a diameter of d=32 mm, Bandyopadhyay et al. [29] reported a heat transfer coefficient of $h_{1}=40,000$ W/m^2^K during quenching and a quenching time of $t_{1}=1.7$ s. For a rebar with a diameter of d=16 mm, the authors reported a heat transfer coefficient of $h_{1}=15,000$ W/m^2^K and a quenching time of $t_{1}=1.3$ s. For a rebar diameter of d=28 mm, linear interpolation yields to a value of $h_{1}=34,000$ W/m^2^K for the heat transfer coefficient and $t_{1}=1.6$ s for the quenching time. However, for the numerical investigations a value of $t_{1}=1.5$ s has been used for the quenching time as this value leads to a correct phase fraction distribution after cooling (see Section 4.2). ^(4)^ With a quenching time of $t_{1}=1.5$ s, the total cooling time results in $t_{1}+t_{2}=3000$ s. For the chosen parameters of the heat transfer coefficients, $h_{1}$ and $h_{2}$, the rebar is cooled down approximately to room temperature (see Section 4.2.4).

**Table 5:** Yield stresses of (nearly) untempered and fully tempered martensite, $Y_{\mathrm{mar}(0)}$ and $Y_{\mathrm{mar}(\infty )}$, as a function of temperature, *T*.

*T* [${}^{\circ }C$]	$Y_{\mathrm{mar}(0)}$ [MPa]	$Y_{\mathrm{mar}(\infty )}$ [MPa]
0	1315	530
200	1065	420 ^(1)^
300	395	388 ^(1)^
600	118	116 ^(1)^

^(1)^ Calculated value.

## Data Availability

Data are contained within the article.
